# Glycogen metabolism-mediated intercellular communication in the tumor microenvironment influences liver cancer prognosis

**DOI:** 10.32604/or.2023.029697

**Published:** 2024-02-06

**Authors:** YANG ZHANG, NANNAN QIN, XIJUN WANG, RUI LIANG, QUAN LIU, RUOYI GENG, TIANXIAO JIANG, YUNFEI LIU, JINWEI LI

**Affiliations:** 1 Graduate School, Kunming Medical University, Kunming, 650000, China; 2 Department of Vascular Surgery, Fuwai Yunnan Cardiovascular Hospital, Affiliated Cardiovascular Hospital of Kunming Medical University, Kunming, 650000, China; 3 Department of Neurosurgery, West China Hospital, Sichuan University, Chengdu, 610000, China; 4 Department of Neurosurgery, The Fourth Affiliated Hospital of Guangxi Medical University, Liuzhou, 545000, China; 5 Department of Gynecology Oncology, The Fourth Affiliated Hospital of Guangxi Medical University, Liuzhou, 545000, China; 6 School of Basic Medical Sciences, Xianning Medical College, Hubei University of Science and Technology, Xianning, 437100, China; 7 College of Bioengineering, Chongqing University, Chongqing, 400030, China; 8 Department of General, Visceral, and Transplant Surgery, Ludwig-Maximilians-University Munich, Munich, 81377, Germany

**Keywords:** Glycogen metabolism, Metabolic map, Single-cell, Tumor microenvironment, Liver cancer, Prognosis, Immunotherapy

## Abstract

Glycogen metabolism plays a key role in the development of hepatocellular carcinoma (HCC), but the function of glycogen metabolism genes in the tumor microenvironment (TME) is still to be elucidated. Single-cell RNA-seq data were obtained from ten HCC tumor samples totaling 64,545 cells, and 65 glycogen metabolism genes were analyzed by a nonnegative matrix factorization (NMF). The prognosis and immune response of new glycogen TME cell clusters were predicted by using HCC and immunotherapy cohorts from public databases. HCC single-cell analysis was divided into fibroblasts, NT T cells, macrophages, endothelial cells, and B cells, which were separately divided into new cell clusters by glycogen metabolism gene annotation. Pseudo-temporal trajectory analysis demonstrated the temporal differentiation trajectory of different glycogen subtype cell clusters. Cellular communication analysis revealed extensive interactions between endothelial cells with glycogen metabolizing TME cell-related subtypes and different glycogen subtype cell clusters. SCENIC analysis of transcription factors upstream of TME cell clusters with different glycogen metabolism. In addition, TME cell clusters of glycogen metabolism were found to be enriched in expression in CAF subtypes, CD8 depleted, M1, and M2 types. Bulk-seq analysis showed the prognostic significance of glycogen metabolism-mediated TME cell clusters in HCC, while a significant immune response was found in the immunotherapy cohort in patients treated with immune checkpoint blockade (ICB), especially for CAFs, T cells, and macrophages. In summary, our study reveals for the first time that glycogen metabolism mediates intercellular communication in the hepatocellular carcinoma microenvironment while elucidating the anti-tumor mechanisms and immune prognostic responses of different subtypes of cell clusters.

## Introduction

Liver cancer is one of the deadliest cancers worldwide. Tumors of hepatic origin include hepatocellular carcinoma, cholangiocarcinoma, and hepatoblastoma [1,2]. Hepatocellular carcinoma (HCC) accounts for 70%–90% of all liver cancer incidences worldwide [3,4]. The development of liver cancer is closely associated with various factors, including hepatitis B and hepatitis C virus infections, aflatoxin, alcohol, cirrhosis, sex hormones, nitrosamines, and trace elements [5]. Therefore, a deeper understanding of the molecular mechanism of HCC may help develop new strategies for the prevention and treatment of HCC.

Metabolism plays an important role in various diseases [6,7]. The liver serves as the central hub for glucose metabolism within the human body. Upon digestion of dietary starch, glucose is produced and absorbed in the intestines. This glucose is subsequently converted into glycogen in two primary sites: the liver and muscles. Glycogen, serving as the principal reservoir for carbohydrate storage in mammals, is distributed across several tissues, including the liver, muscle, kidney, and brain [8,9]. Glycogen synthesis and degradation consume or produce glucose-6-phosphate (G6P), a key metabolite necessary for central carbon metabolism [10]. Furthermore, it is postulated that immune cells predominantly enhance activation-driven glycolysis through upregulated expression of glucose transporter proteins [11,12]. Despite extensive documentation on the reciprocal regulations between the liver and gluconeogenesis, our current understanding of the molecular landscape governing glycogen metabolism in hepatocellular carcinoma remains limited.

The tumor microenvironment (TME) represents a complex interplay among tumor cells, immune cells, inflammatory cells, and tumor-associated fibroblasts. Tumors exert control over the TME by promoting angiogenesis, establishing metabolic symbiosis with stromal cells, and inducing peripheral immune tolerance [13–15]. In turn, immune cells within the microenvironment exert influence over the proliferation and evolutionary trajectory of cancer cells. Thwe et al. reported that inhibition of glycogenolysis significantly attenuated toll-like receptor-mediated dendritic cell maturation and impaired its ability to initiate lymphocyte activation [16]. Immune responses include rapid and widespread changes in immune cell activity, accompanied by changes in cellular metabolism. These metabolic shifts are driven by nutrients, including glucose, amino acids, and fatty acids, and significantly impact the fate and function of immune cells [17]. Therefore, unraveling the intricacies of cellular metabolism and deciphering the activation states of distinct immune subpopulations are crucial for gaining insights into the TME.

To understand the relevance of glycogen metabolism within the TME of HCC, we conducted a comprehensive investigation on the impact of glycogen metabolism on various cell types, including stromal, myeloid, T and B cells, and tumor-associated fibroblasts. Leveraging 71,915 single-cell sequencing data obtained from ten HCC patients, we employed non-negative matrix factorization (NMF) to identify distinct subpopulations of TME cell types in HCC. By exploring the association between newly identified glycogen-metabolizing cell subtypes and diverse immune characteristics, metabolic pathways, cellular interactions, and prognosis in HCC, we aimed to provide a comprehensive single-cell analysis of glycogen metabolism. Such analysis holds potential in unraveling the intricate interplay between the TME and tumor cells, ultimately facilitating novel staging and prognostication approaches for HCC.

## Materials and Methods

### Data collection

We collected and analyzed single-cell transcriptome sequencing data from four relevant tissue types, namely non-tumor liver, primary tumor, portal vein thrombosis, and metastatic lymph node tissue, obtained from ten hepatocellular carcinoma (HCC) patients in the GSE149614 dataset. Primary tumors and non-tumor liver tissue were selected for further analysis. Transcriptomic data and clinical prognosis-related data were collected from The Cancer Genome Atlas (TCGA) for 424 HCCs. To validate our findings, we utilized transcriptomic data from 240 HCC samples in the International Cancer Genome Consortium (ICGC) liver cancer cohort (LIRI). We collected a total of 14 metabolic pathways and 94 genes related to glycogen metabolism from the MSigDB database [18,19].

### Visualization of TME cell types in HCC

To visualize the TME cell types in HCC, we employed the R package “Seurat” to create Seurat objects based on the single-cell RNA sequencing (scRNA-seq) gene expression matrix of HCC [20]. A total of 13,714 genes and 2,700 cells underwent quality control procedures. We filtered out low-quality cells by removing those with less than 200 unique molecular identifiers (UMIs) and those with gene expression levels exceeding 8,000 or falling below 200 cells (Suppl. Fig. 1A). Additionally, we eliminated dead or dying cells by removing those with more than 10% UMIs originating from the mitochondrial genome. Consequently, we obtained a total of 71,915 high-quality single-cell transcriptomes across all samples. For dimensionality reduction, we selected a scale matrix comprising 2,000 highly variable genes. To integrate tissue and patient samples, we employed the R package “harmony” for data scaling and integration (Suppl. Fig. 1B). UMAP/tSNE was used for data visualization, and cellular subpopulations were annotated using previously reported marker genes (Suppl. Figs. 1C and 1D) [21].

### Pseudo-temporal trajectory analysis of glycogen metabolism genes in TME cells

To investigate single cell trajectory analysis with glycogen metabolism in HCC TME, we used the Monocle R package to perform developmental sorting of all subtypes of cells in HCC. Analysis criteria: mean expression >= 0.1 & dispersion empirical >= 1 * dispersion fit. Data downscaling was performed using the ‘DDRTree’ method. The distribution of glycogen metabolism was visualized in the NMF cluster developmental trajectory of different TME cells.

### Non-negative matrix factorization of glycogen metabolizing genes in TME cells

To investigate the NMF of 94 glycogen metabolism genes on different TME cells, we constructed an expression matrix of glycogen metabolism genes. Cells that did not express any glycogen metabolism genes were removed. The matrix decomposition was performed using the “NMF” package, followed by dimensional reduction clustering. These methods are similar to those reported in previous studies by Chen et al. and Puram et al. [22,23].

### Identification of cellular subtype marker genes associated with glycogen metabolism genes in TME cells

To further identify NMF subsets of cells, we reannotated them based on specific criteria. Annotation was performed by identifying characteristic genes for each cluster. The annotation criteria were as follows: 1) logFC > 1, with the top-ranked signature gene being a glycogen metabolism gene; 2) logFC > 1, but without glycogen metabolism gene expression, indicating non-glycogen-related genes; 3) logFC < 1, with unclear relation to glycogen metabolism genes.

### Functional enrichment analysis of NMF glycogen metabolism-related subtypes

To explore the metabolic characteristics of glycogen metabolism-related subtypes identified through NMF typing, we conducted an enrichment score analysis. We employed the “metabolism” function to randomly generate 30 metabolic signaling pathway scores for demonstration purposes.

### Cell communication analysis for NMF glycogen metabolism-related subtypes of cells

To further identify the cellular interactions between NMF glycogen metabolizing cell types, we performed intercellular ligand-receptor analysis using the CellChat function [24]. First, we extracted NMF glycogen cell subtypes and immune cells and then identified overexpressed genes and ligand-receptor pairs. Finally, the ligands and receptors were projected to the Protein-Protein Interaction (PPI) network for weight analysis. The outgoing and incoming signaling pathways of different MNDs were also visualized.

### SCENIC analysis for glycogen metabolism-related subtypes

Transcription factors (TFs) are a group of proteins that act directly on the genome, bind to specific DNA sequences (TFBS/motif), and regulate the process of DNA transcription. Cell communication analysis was performed for NMF glycogen metabolism-related subtypes. Transcription start sites (TSS) and gene regulatory networks in scRNA-seq data in HCC were examined using gene motif ranking (hg19-tss-centered-10 kb-7 species) from the RcisTarget database. Firstly, the default parameters were used for gene filtering and the filtering criteria were the sum of gene expression > cell number * 3% and expressed in 1% of cells. Subsequently, the correlation matrix was calculated. Next, TF-Targets correlation regression analysis was performed. Due to a large number of cell types, the Regulon Specificity Score (RSS) was used to identify cell type-specific regulons. A z-score > 1.5 was selected to further map the transcription factor FeaturePlot. Regulon scores heatmap was also plotted. Further visualization was performed by using the “ComplexHeatmap”, “ggplot2”, and “pheatmap” packages [18,25].

### Prognostic analysis of glycogen metabolism-related genes for HCC

To evaluate the prognostic implications of glycogen metabolism-related subtypes of cells in HCC at the bulk level, we calculated genetic marker scores in publicly available HCC datasets using the “GSVA” function [26]. We used optimal Cutoff, log-rank test, and Cox proportional risk regression to explore the association between glycogen metabolism-related NMF characteristics and overall patient survival (OS). Kaplan-Meier curves were plotted using the “survminer” R package for different cell subtypes [19]. Additionally, we visualized the prognosis of glycogen metabolism-related NMF subtypes across different datasets using the “ggplot” function.

### Immunotherapeutic response of NMF glycogen metabolizing subtype cells

We predicted the immunotherapeutic responses of NMF glycogen metabolism-related subtypes using the TIDE database, which estimates patient response based on the expression profiles prior to tumor treatment using multiple published transcriptional biomarkers [27]. We utilized the LIRI and LIHC transcriptomic datasets from the TIDE database to explore the immune response of NMF glycogen metabolic subtypes.

### Statistical analysis

To compare differences between subgroups of different subtypes, we employed Student’s *t*-test, Wilcoxon rank-sum, and Kruskal-Wallis tests. We determined TME-related genes and subtypes of cancer-associated fibroblasts (CAFs) for correlation analysis with subtype cells. All statistical analyses were performed using R 4.1.2 software, and *p*-values below 0.05 were considered statistically significant.

## Results

### Landscape of glycogen metabolism genes in TME cells in HCC

**FIGURE 1:**
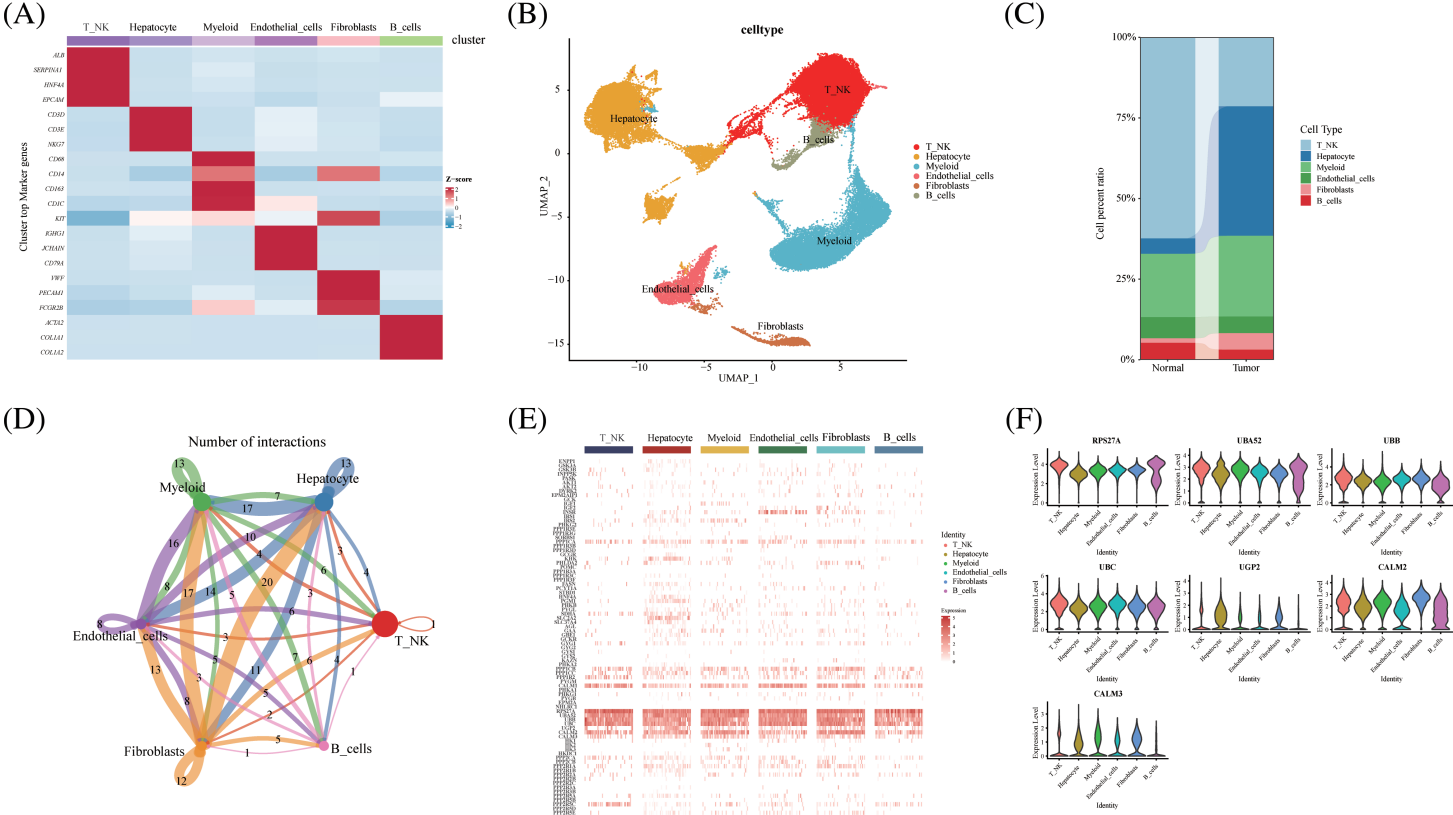
Overview of glycogen metabolism-related genes in hepatocellular carcinoma single-cell data. (A) Heat map showing marker genes annotating subpopulations of hepatocellular carcinoma cells. (B) The UMAP visualizes six cell subpopulations of hepatocellular carcinoma cells. (C) Cell percentage diagram showing the proportion of cell subpopulations in normal and hepatocellular carcinoma tissues. (D) CellChat analysis showing cellular interactions of subpopulations of hepatocellular carcinoma cells. (E) Distribution of glycogen metabolism-related genes in the heat map of NK T cells, hepatocyte cells, myeloid cells, endothelial cells, fibroblasts, and B cells. (F) Violin diagram showing the expression of glycogen metabolism genes in TME subpopulation cells.

We further explored glycogen metabolism-related genes in the HCC landscape map, by using single-cell data from ten tumor tissues and their adjacent tissues, two liver cancer prognosis datasets, and an immunotherapy dataset. We performed cellular annotation by marker genes from the original literature, mainly for the following cells: NKT Cells, hepatocyte cells, myeloid cells, endothelial cells, fibroblast cells, and B cells (Figs. 1A and 1B). The proportions of different cell types varied between tumor tissues and normal tissues, such as fibroblasts and hepatocytes (Fig. 1C). CellChat analysis showed interactions between individual subtypes of cells (Fig. 1D). We then visualized the expression of 94 genes related to glycogen metabolism in different components of TME cells using a heat map (Fig. 1E). Violin plots demonstrated that RPS27A, UBA52, UBB, UBC, UGP2, CALM2, and CALM3 genes were highly expressed in a variety of cells (Fig. 1F). The expression of glucose metabolism genes may be involved in intercellular interactions in the tumor microenvironment.

### Novel glycogen metabolizing CAF subtype cells from TME of HCC

**FIGURE 2:**
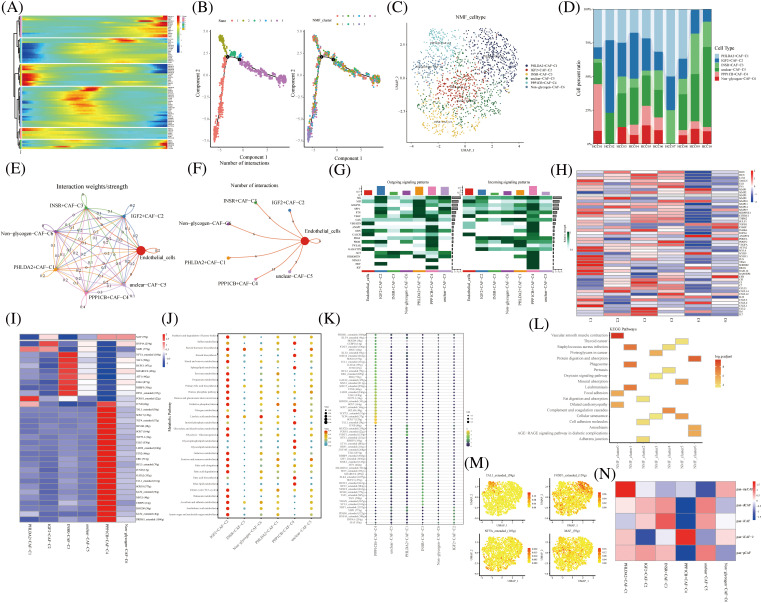
Novel fibroblast clusters under gluconeogenic gene modifications. (A) Trajectory analysis reveals the role of glycogen metabolism genes in fibroblast clustering after NMF classification. (B) Pseudo time shows the differentiation of fibroblast clusters after NMF classification. (C) UMAP shows fibroblast clusters after glycogen gene annotation. (D) Cell percentages shows expression of novel fibroblast clusters in ten HCC patients. (E and F) CellChat analysis reveals the interaction of novel fibroblast clusters with endothelial cells. (G) The signaling pathways of the novel fibroblast cluster inputs and outputs are shown by a heat map. (H) Expression of immune-related genes in novel fibroblast clusters is shown by a heat map. (I) Active expression of transcription factors in a novel cell cluster of glycogen metabolic fibroblasts. (J) Enrichment of metabolic signaling pathways in glycogen metabolizing fibroblast clusters is shown by a dot plot. (K) Heat map shows the activity of transcription factors in activated novel CAF cell subsets. (L) KEGG enrichment analysis of glycogen metabolizing fibroblast cluster enrichment. (M) Visualization of the expression of transcription factors in a novel cell cluster of glycogen metabolic fibroblasts by UMAP. (N) Heat map demonstrating glycogen metabolizing fibroblast clusters enriched in a characteristic subpopulation of previous fibroblasts.

Pseudo-temporal analysis revealed that glycogen metabolism-related genes play a key role in the trajectory of TME cells, including NKT Cells, hepatocyte cells, myeloid cells, endothelial cells, fibroblasts, and B cells (Fig. 2A). Previous literature has shown that metabolic competition in tumor microenvironment affects immune cell metabolism [28]. We found that the different states or clusters are divided into three branches (Fig. 2B). CAF subtype cells were subjected to NMF and divided into the following subtypes by expression of glycogen metabolism genes: PHLDA2+CAF-C1, IGF2+CAF-C2, INSR+CAF-C3, unclear-CAF-C5, PPP1CB+CAF-C4 and Non-glycogen-CAF-C6 (Figs. 2C and 2D). CellChat analysis revealed different interactions of novel CAF subtypes of cells (Fig. 2E). Fibroblasts are an important part of tumor stroma. CAF and acidified microenvironment drive tumor cells to spontaneously choose glucose metabolism and promote tumor malignant progression [29]. Therefore, the labeling of different glucose metabolic cells is helpful to identify the interrelation and coordination of glucose metabolic fibroblasts. Meanwhile, endothelial cells interacted most with PPP1CB+CAF-C4 cell clusters (Fig. 2F). In addition, PPP1CB+CAF-C4 cell clusters were found to be associated with most Outgoing signaling patterns and Incoming signaling patterns (Fig. 2G). The signals contributing most to the output and input signals of the PHLDA2+CAF-C1 cell cluster were MIF, ANGPTL, SPP1, PTN, GRN, and PERIOSTIN. The signals that contributed most to the output or input signals of IGF2+CAF-C2 cell clusters were MK, MIF, PTN, and PROS. The signals that contributed most to the output and input signals of the INSR+CAF-C3 cell cluster were MK, SPP1, and GAS. Signals contributing to the output and input signals of the PPP1CB+CAF-C4 cell cluster were MK, MIF, ANGPTL, PTN, VEGF, GAS, ANGPT, GRN, PDGF, PROS, TWEAK, SEMA3, and IGF. These are related signal pathways involved in tumor growth, proliferation and inhibition. Similarly, the signals that contributed most to the output and input signals of the unclear-CAF-C5 cell cluster were MK, SPP1, PTN, GAS and Tet signals that contributed most to the output and input signals of the non-glycogen-CAF-C6 cell cluster were PTN, VEGF, and PDGF. In addition, the expression of immune-related genes in novel CAF cell clusters was observed and most of them were found to be expressed in IGF2+CAF-C2 cell clusters (Fig. 2H). The activity of most transcription factors was strong in the non-glycogen-CAF-C6 cell cluster (Figs. 2I) IGF2+CAF-C2 cell clusters were found to be enriched in metabolic signaling pathways such as glyoxylate and dicarboxylate metabolism, glycolysis/Collins. gluconeogenesis, galactose metabolism, fructose, and mannose metabolism, fatty acid elongation and amino sugar and nucleotide metabolism (Fig. 2J). High expression of transcription factors in the PPP1CB+CAF-C4 cell cluster (Fig. 2K).

It was also demonstrated that IGF-binding proteins secreted by cancer-associated fibroblasts induce cancer cell-dependent drug sensitization [30]. This suggests that IGF-related genes promote the involvement of fibroblasts in cancer progression.

PHLDA2+CAF-C1 cell clusters are enriched in the signaling pathway of vascular smooth muscle contraction (Fig. 2L). High expression of transcription factors TAL1, FOXF1, NFYA, and MAF in CAF subtype cell clusters is shown in UAMP plots (Figs. 2M).

Typing-related gene marker, pan-myCAF, was concentrated in the PHLDA2+CAF-C1 cell cluster expression. Similarly, pan-dCAF typing-related gene marker is concentrated in unclear-CAF-C5 cell cluster expression and pan-iCAF typing-related gene marker is concentrated in the INSR+CAF-C3 cell cluster expression. In addition, pan-iCAF-2 and pan-pCAF typing-related gene markers were concentrated in the PPP1CB+CAF-C4 cell cluster expression And in IGF2+CAF-C2 and unclear-CAF-C5 cell cluster expression, respectively (Fig. 2N). Thus, our labeled fibroblast clusters of glycolytic metabolism can correspond to some extent to the classical fiber phenotype [31].

### Novel glycogen metabolizing CD8+ T subtype cells for TME of HCC

**FIGURE 3:**
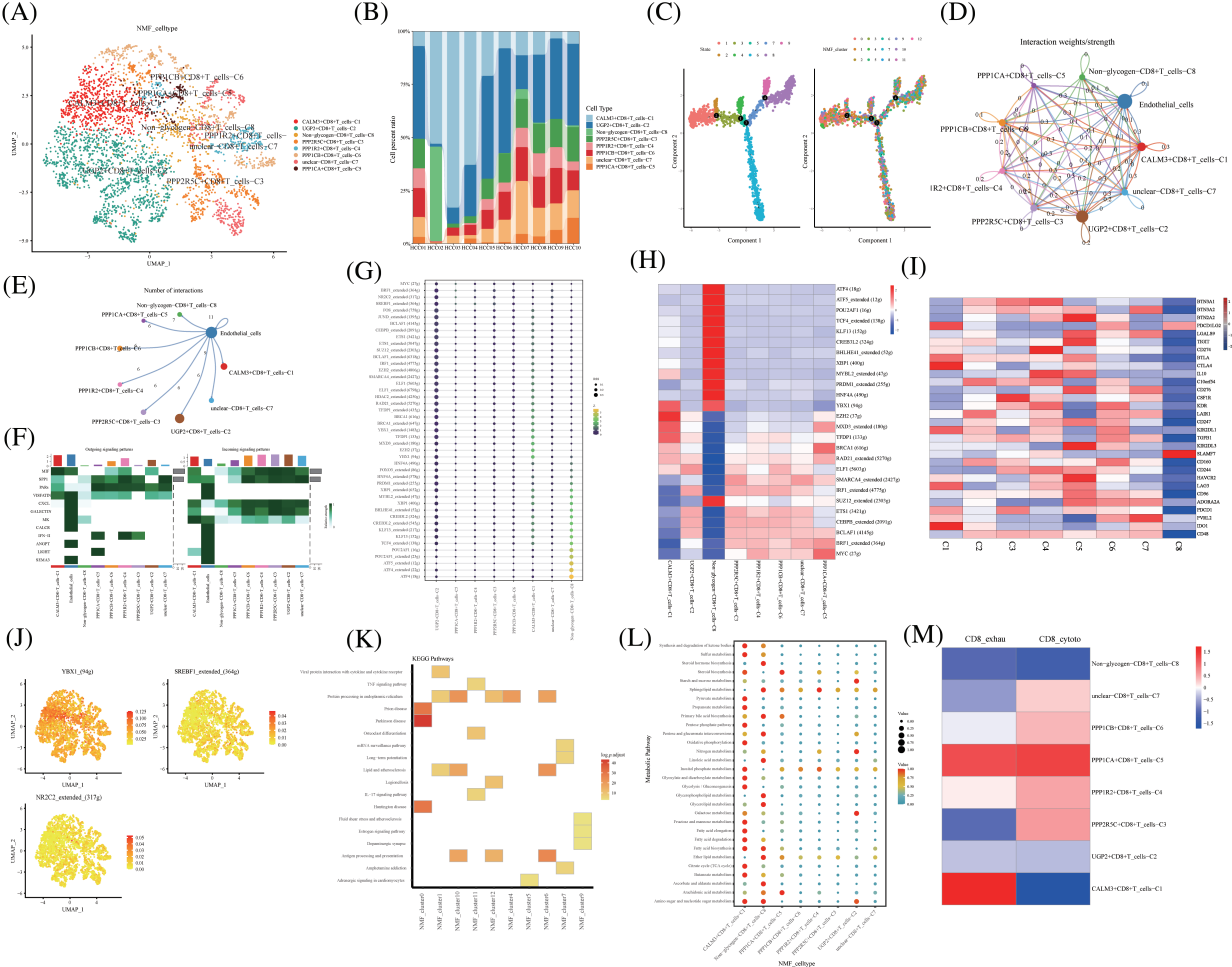
Novel CD8+ T cell clusters under gluconeogenic gene modifications. (A) UMAP demonstrates CD8+ T cell clustering fractionation after glycogen metabolism annotation. (B) Cell percentage plots demonstrate the percentage of expression of novel glycogen metabolizing CD8+ T cell clusters. (C) Pseudo time demonstrates the differentiation of CD8+ T cells after NMF classification. (D and E) Cell Chat shows the interaction of novel CD8+ T cell clusters with endothelial cells. (F) The signaling pathways of the novel CD8+ T cell cluster inputs and outputs are shown by a heat map. (G and H) Transcription factor activities of glycogen metabolizing CD8+ T cell clusters are shown by bubble plots, and heat maps, respectively. (I) Expression of immune-related genes in novel CD8+ T cell clusters is shown by a heat map. (J) UMAP shows expression of transcription factors for glycogen metabolizing CD8+ T cell clusters. (K) KEGG enrichment analysis of glycogen metabolizing CD8+ T cell cluster enrichment. (L) Enrichment of metabolic signaling pathways in glycogen metabolizing CD8+ T cell clusters is shown by a dot plot. (M) Heat map demonstrating novel glycogen metabolizing CD8+ T cell subtype cells correlated with CD8+ T cell depletion and activation.

The NK T cells were further divided into subpopulations of cells annotated as CD4+ T, CD8+ T, Treg, and unknown cells. CD8+ T cells were divided into eight cell clusters by NMF, annotated by glycogen metabolism genes: CALM3+CD8+T cells-C1, UGP2+CD8+T cells-C2, Non-glycogen-CD8+T cells-C8, PPP2R5C+CD8+T cells-C3, PPP1R2+CD8+T cells-C4, PPP1CA+CD8+T cells-C5, PPP1CB+CD8+T cells-C6, and unclear-CD8+T cells-C7 (Fig. 3A). The proportion of CD8+ T cell cluster types with glycogen metabolism were differentially expressed in ten HCC patients (Fig. 3B). The novel CD8+ T cell cluster was divided into four branches by a proposed temporal sequence (Fig. 3C). Meanwhile, CellChat showed the strength of the correlation between novel glycogen metabolizing CD8+ T cell clusters (Fig. 3D). Glucose metabolism is the main metabolic pathway required after T cell activation, and glucose restriction in TME significantly affects T cell responses. It has been previously reported that increasing the glycolytic capacity of mouse sarcoma cells in co-culture experiments can lead to suppression of CD8+ T cell effector functions. By analyzing the interaction of different novel glycogen-metabolizing CD8+ T cells with endothelial cells, the highest number of CALM3+CD8+T cells-C1 interactions was observed (Fig. 3E). CellChat signaling analysis revealed that the incoming signaling patterns of CALM3+CD8+ T cells-C1 cell clusters were mainly in the IMK, GALECTIN, CXCL, SPP1, and MIF signaling pathways. The SPP1, PARs, MIF and VISFATIN signaling pathways served as the main outgoing signaling patterns involved in the novel glycogen metabolizing CD8+ T subtype (Fig. 3F). Higher transcription factor activity was found in the Non-glycogen-CD8+T cells-C8 cell cluster (Figs. 3G and 3H). Next, novel CD8+ T cell subpopulation analysis of key immune checkpoint genes revealed that the Non-glycogen-CD8+ T cells-C8 cell cluster was negatively associated with most of the immune checkpoint-related genes (Fig. 3I). Increased glucose uptake is one of the most common features of malignancy. Most cancer cells prefer to rely on glycolysis (also known as the Warburg effect) for proliferation and survival, while aerobic glycolysis is also required for T cell activation, differentiation and effector functions.

Previous studies have demonstrated that the CTLA-4 pathway competitively inhibits CD28-mediated co-stimulation and reduces Akt phosphorylation and activation, thereby impairing T cell glycolysis and mitochondrial remodeling. Moreover, interactions between immune checkpoints and their ligands (e.g., PD-1/PD-L1 and CTLA-4/CD86) are further involved in the metabolic reprogramming of tumor cells and immune cells [32]. The PPP1CA+CD8+T cells-C5 cell cluster was positively correlated with most of the immune checkpoint-related genes (BTN2A2, LGALS9, TIGIT, BTLA, CTLA4, IL10C10orf54, CD276, CSF1R, KDR, LAIR1, CD247, KIR2DL1, and TGFB1) (Fig. 3I). Transcription factors can regulate the expression of downstream target genes and serve as important regulators for maintaining cellular properties. Therefore we identified transcription factors specific to the CD8+ T cell glycation subtype cell cluster. Transcription factors YBX1, SREBF1, and NR2C2 were highly expressed in CD8+ T cells glycolytic subtype cell clusters (Figs. 3J). Most of the cell clusters were enriched in protein processing in the endoplasmic reticulum signaling pathway (Fig. 3K). CALM3+CD8+ T cells-C1 cell clusters are enriched in most metabolic signaling pathways (Fig. 3L). CD8+ T cell depletion-related genes are mainly expressed in the CALM3+CD8+T cells-C1 cell cluster (Fig. 3M). The cytotoxic-related genes of CD8+ T cells were mainly expressed in PPP2R5C+CD8+T cells-C3, PPP1R2+CD8+T cells-C4, PPP1CB+CD8+T cells-C6, and unclear-CD8+T cells-C7 cell clusters (Fig. 3M).

### Novel glycogen metabolizing B subtype cells for TME of HCC

**FIGURE 4:**
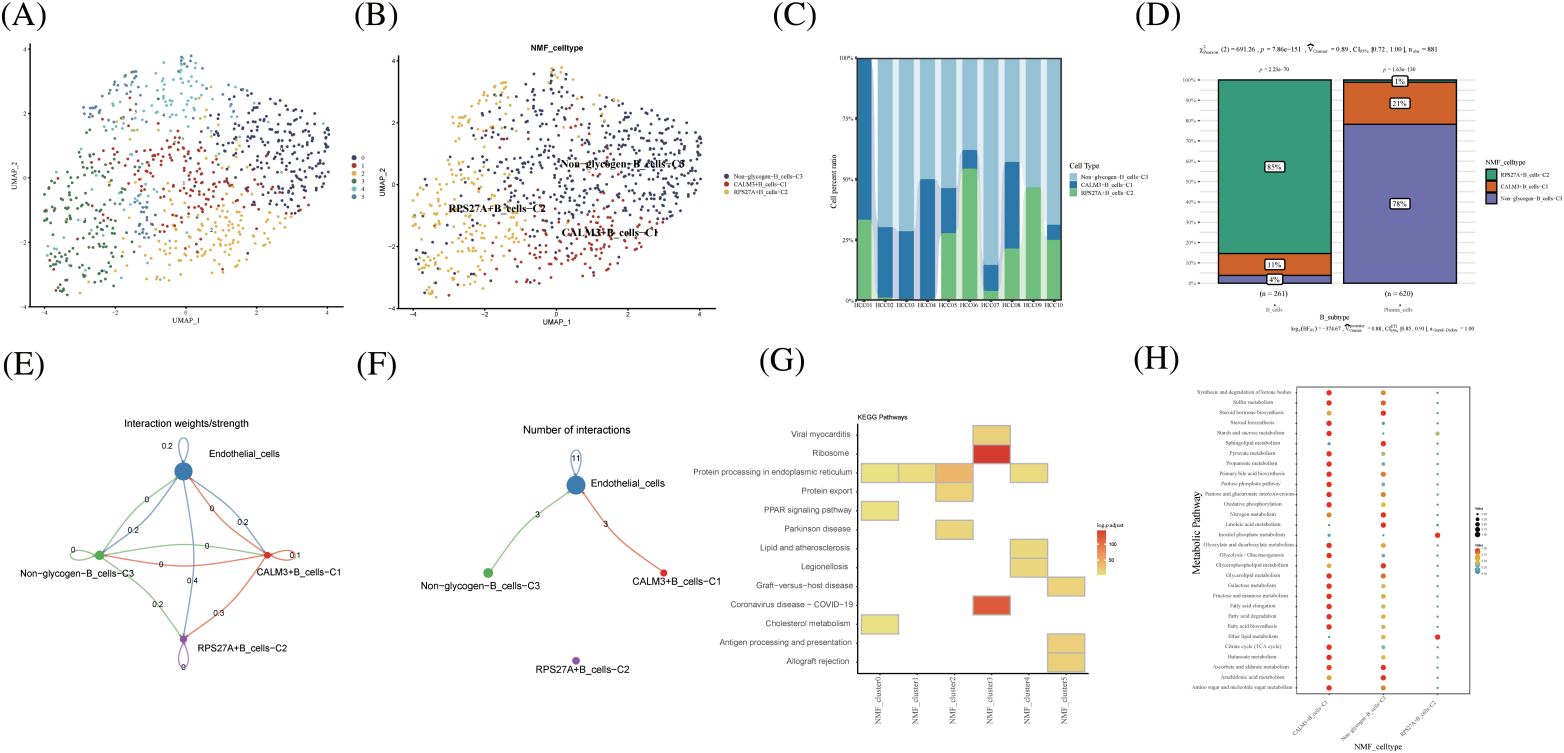
Novel B cell clusters under gluconeogenic gene modifications. (A) UMAP demonstrates new cell clusters of B cells after clustering by NMF. (B) UMAP demonstrates novel B cell clusters via glycogen metabolism gene markers. (C) Cell percentage plots demonstrate the expression of novel glycogen metabolizing B cell clusters in patients with hepatocellular carcinoma. (D) Cell percentage plots demonstrate the expression of novel glycogen metabolizing B cell clusters and plasma cells in patients with hepatocellular carcinoma. (E and F) Cell Chat reveals the interaction of novel B cell clusters with endothelial cells. (G) KEGG enrichment analysis of glycogen metabolizing B cell cluster enrichment. (H) Enrichment of metabolic signaling pathways in glycogen metabolizing B cell clusters is shown by a dot plot.

As a key component of the immune system, B cells play an important role in antibody production, immune regulation and memory response. B cells require high energy metabolic pathways to support their functions and activities. Among them, glucose metabolism, as a major energy source and regulator, plays an important role in the physiological and pathological processes of B cells. B cells were divided into 5 cell clusters after clustering by NMF (Fig. 4A). Using genes related to glycogen metabolism, B cells were further divided into CALM3+B cells-C1, RPS27A+B cells-C2, and Non-glycogen-B cells-C3 (Fig. 4B). RPS27A+B cells-C2 cell clusters occupied 85% of the B cell population (Figs. 4C and 4D). CellChat analysis revealed that the novel B cell subtypes interacted with endothelial cells, mainly in CALM3+B cells-C1 and Non-glycogen-B cells-C3 cell clusters (Figs. 4E and 4F). Meanwhile, NMF cell cluster 3 was mainly enriched in the Ribosome signaling pathway (Fig. 4G). CALM3+B cells-C1 and Non-glycogen-B cells-C3 cell clusters are enriched with numerous metabolic pathways (Fig. 4H).

### Novel glycogen metabolizing macrophages subtype cells for TME of HCC

**FIGURE 5:**
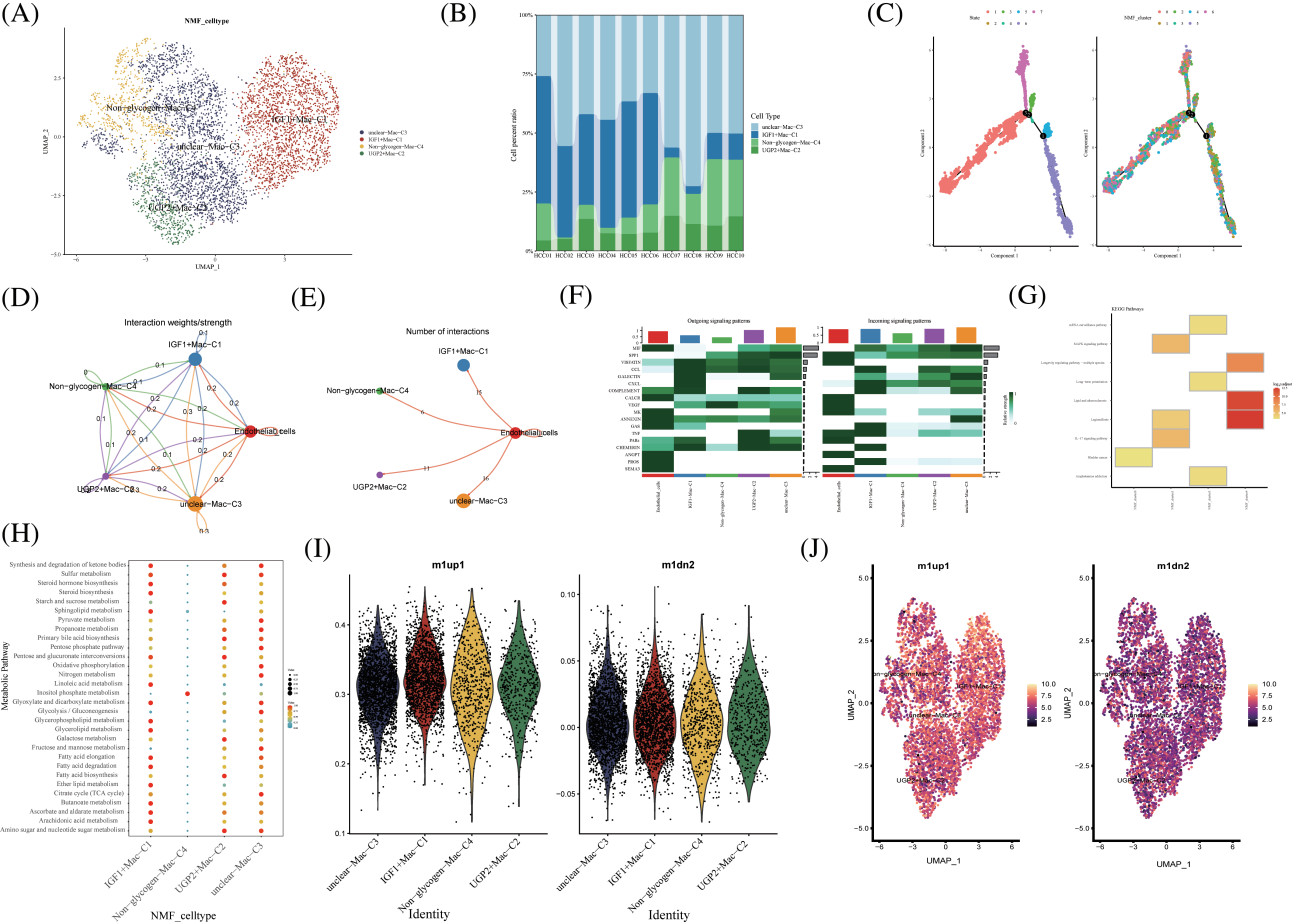
Novel Macrophage cell clusters under gluconeogenic gene modifications. (A) UMAP demonstrates novel glycogen-metabolizing macrophage clusters after clustering by NMF. (B) Cell percentage plots demonstrate the expression of novel glycogen metabolizing Macrophage cell clusters in patients with hepatocellular carcinoma. (C) Pseudo time demonstrates the differentiation of Macrophage cells after NMF classification. (D and E) CellChat reveals the interaction of novel Macrophage cell clusters with endothelial cells. (F) The signaling pathways of the novel Macrophage T cell cluster inputs and outputs are shown by a heat map. (G) KEGG enrichment analysis of Macrophage cell cluster enrichment. (H) Enrichment of metabolic signaling pathways in glycogen metabolizing Macrophage cell clusters is shown by a dot plot. (I) Violin diagram showing the expression of novel glycogen-metabolizing macrophage clusters in M1 and M2 phenotypes. (J) UMAP demonstrates expression of novel glycogen-metabolizing macrophage clusters in M1 and M2 phenotypes.

In hepatocellular carcinoma tissue, macrophages exist as tumor-associated macrophages (TAMs). The number and activity of TAMs are closely related to the progression and prognosis of hepatocellular carcinoma. The novel macrophages were clustered by NMF and annotated by glycogen metabolism genes into the following cell clusters: IGF1+Mac-C1, UGP2+Mac-C2, unclear-Mac-C3, and Non -glycogen-Mac-C4 (Fig. 5A). Among them, the IGF1+Mac-C1 cell cluster occupied the majority of macrophage numbers (Fig. 5B). Pseudo-time analysis of cell clusters was divided into 4 branches (Fig. 5C). CellChat shows interactions between cell clusters of novel glycogen-metabolizing B cell subpopulations (Fig. 5D).

Meanwhile, the highest number of IGF1+Mac-C1 was found to interact with endothelial cells at 15 (Fig. 5E). CXCL as Outgoing signaling patterns was mainly involved in IGF1+Mac-C1 cell clusters. PROS, CHEMERIN and TNF as Incoming signaling patterns are mainly involved in IGF1+Mac-C1 cell clusters (Fig. 5F). We can find that NMF cluster 4 was mainly enriched in Legionellosis, Longevity regulating pathway, and Lipid and atherosclerosis (Fig. 5E). IGF1+Mac-C1, UGP2+Mac-C2, and unclear-Mac-C3 are all enriched in most metabolism-related signaling pathways (Figs. 5G and 5H). Then, we performed a comparison of novel glycogenic macrophage subpopulations in relation to M1 and M2 macrophage typing. As shown in the Figs. 5I–5G, the novel glycogen macrophage cell clusters are mainly concentrated with the M1-type cell subpopulation (Figs. 5I and 5J). TAMs in hepatocellular carcinoma tissues have specific functions and phenotypes, which include features of M2-type macrophages that exhibit immunosuppressive, inflammatory regulatory and fibrosis-promoting properties. TAMs can enhance the activity of gluconeogenic pathways by secreting cytokines and growth factors that promote sugar uptake, oxidative phosphorylation and gluconeogenesis in hepatocellular carcinoma cells [33].

### Prognostic and immunological effects of novel glycogen metabolizing cell clusters on HCC

**FIGURE 6:**
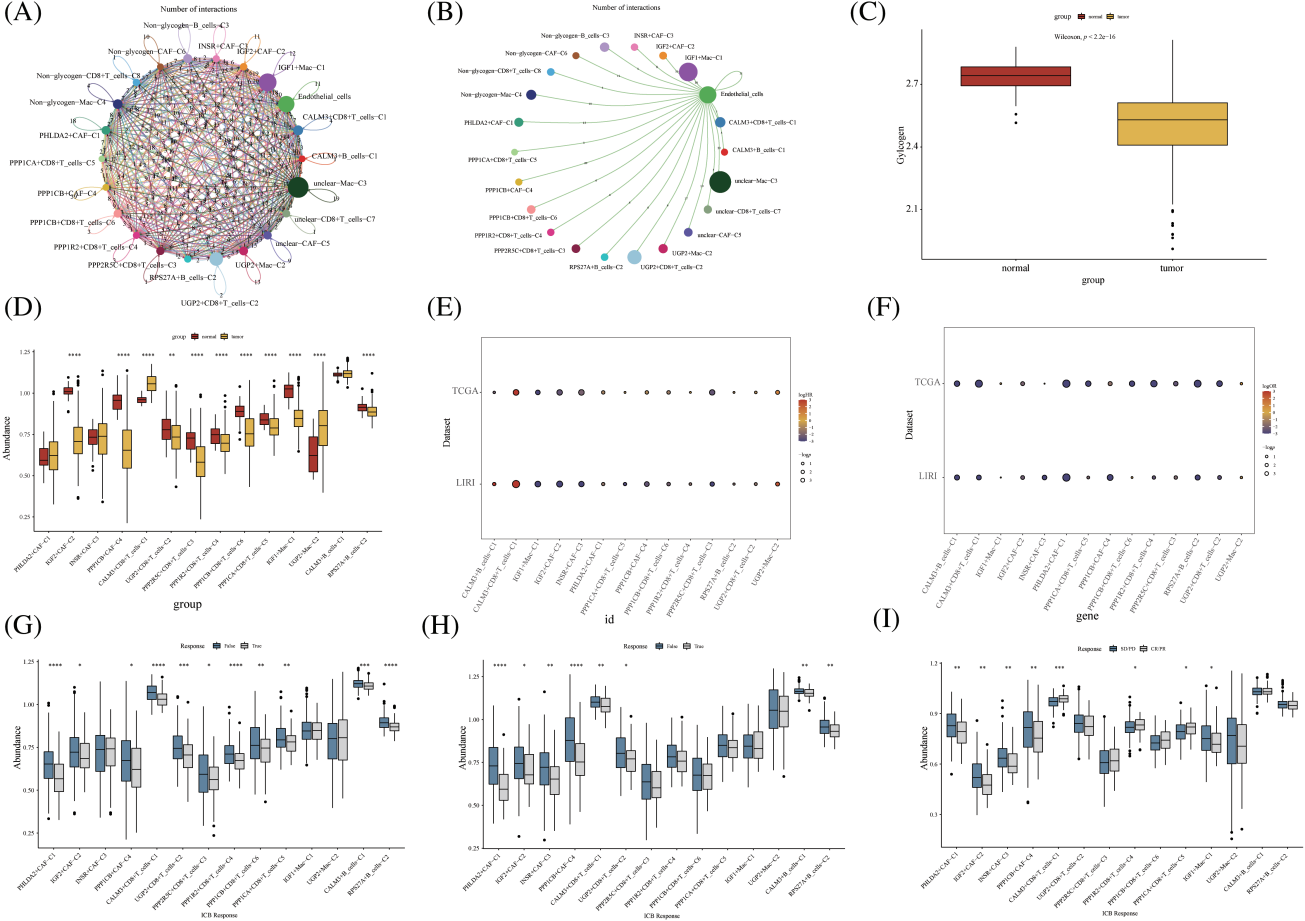
Novel glycogen metabolizing cell cluster prognosis and immune response in patients with hepatocellular carcinoma. (A) Cell Chat demonstrates interactions between all novel glycogen metabolizing cell clusters. (B) Cell Chat demonstrates the interaction between all novel glycogen metabolizing cell clusters and endothelial cells. (C) Box plot showing the difference in glycogen metabolism gene expression in HCC tumor tissue and normal tissue. (D) Box plot demonstrating the difference in expression of novel glycogen metabolizing cell clusters in HCC tumor tissues and normal tissues. (E) Bubble plot demonstrating one-way Cox analysis of novel glycogenic cell clusters in the prognosis of TCGA and LIRI patient cohorts. (F) Bubble plot demonstrating one-way Cox analysis of novel glycogen cell clusters in TCGA and LIRI patient cohort immune response prognosis. (G–I) Box plot demonstrating novel glycogen cell clusters in immune response in TCGA, LIRI, and IMvigor210 patient cohorts. *p < 0.05, **p < 0.01, ***p < 0.001, ****p < 0.0001.

**FIGURE 7:**
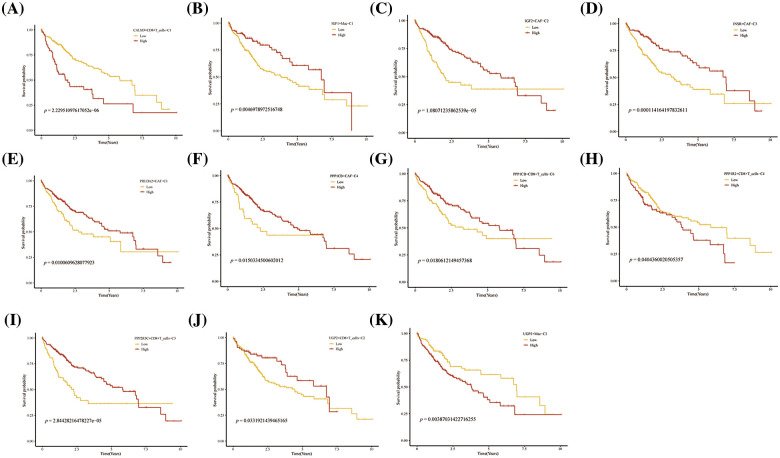
Prognosis of novel glycogen metabolizing cell clusters in the TCGA cohort.

**FIGURE 8:**
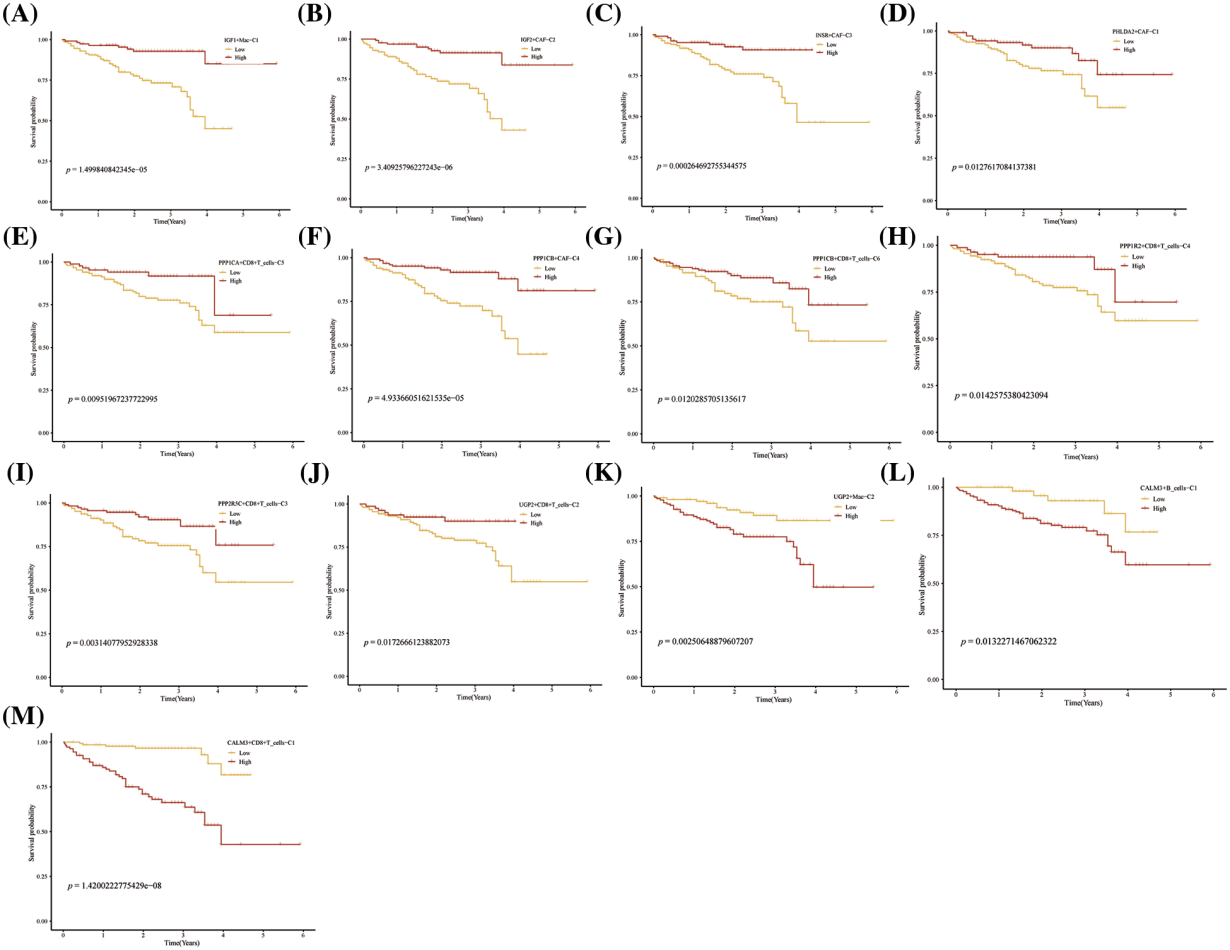
Prognosis of novel glycogen metabolizing cell clusters in the LIRI cohort.

**FIGURE 9:**
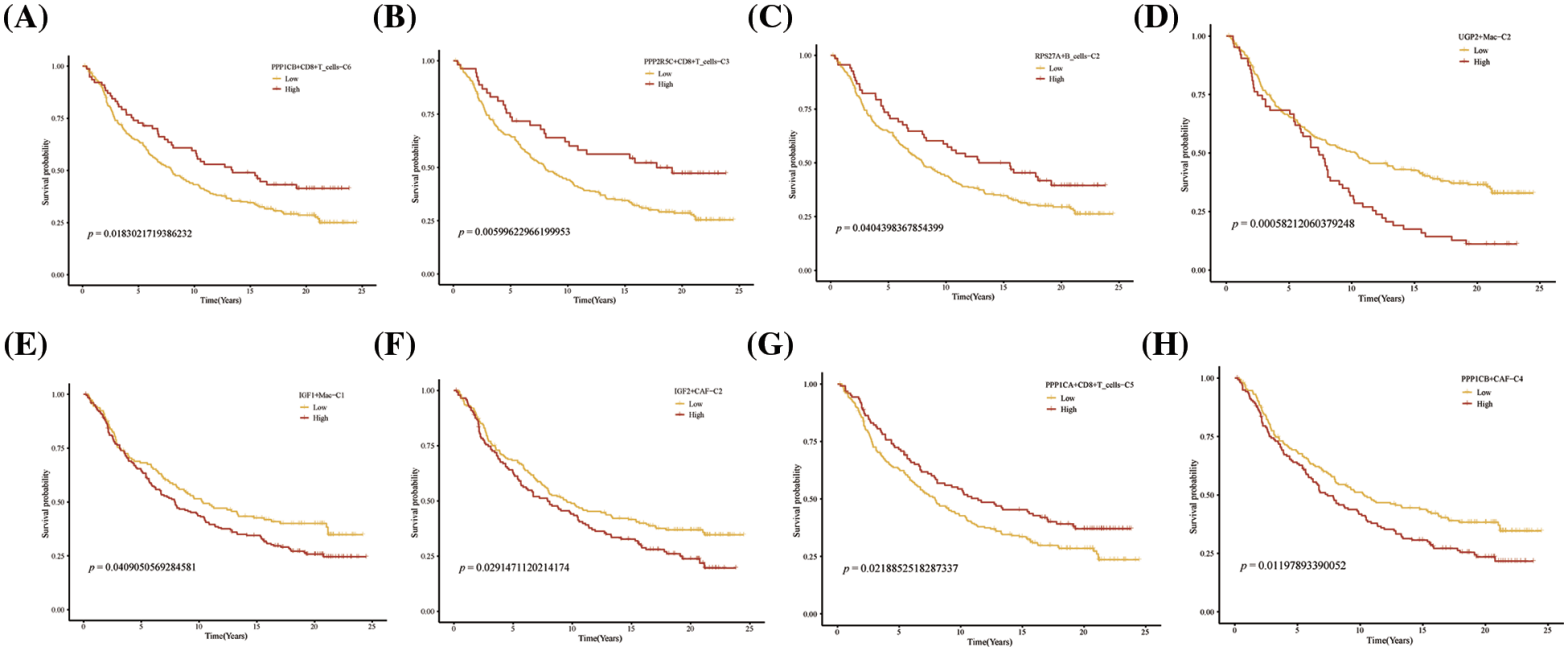
Prognosis of novel glycogen metabolizing cell clusters in the IMvigor210 cohort.

Further, we performed the prognosis of the subtype cells by transcriptional and prognostic information of TGCA and LIRI liver cancer cohorts. CellChat indicated that all glycogen metabolizing subtypes of cells in HCC exist to interact with each other (Fig. 6A). PPP1CB+CAF-C4, UGP2+Mac-C2, and unclear-Mac-C3 cell clusters interacted more with endothelial cells (Fig. 6B). The box plot demonstrates that glycogen-related genes are less expressed in tumor tissues than in normal tissues in the LICH population (Fig. 6C). Meanwhile, we found that the cell types that were lowly expressed in tumor tissues compared with normal tissues were IGF2+CAF-C2, PPP1CB+CAF-C4, UGP2+CD8+T cells-C2, PPP2R5C+CD8+T cells-C3, PPP1R2+CD8+T cells-C4, PPP1CB+CD8+T cells-C6, PPP1CA+CD8+T cells-C5, IGF1+Mac-C1 and RPS27A+B cells-C2 (*p* < 0.05) (Fig. 6D). Cell types that are highly expressed in tumor tissues compared to normal tissues are CALM3+CD8+T cells-C1 and UGP2+Mac-C2 (*p* < 0.05) (Fig. 6D). Subsequently, univariate Cox analysis revealed a consistent prognosis for different novel glycogen-metabolizing subtypes of cells in different datasets (Fig. 6E). CALM3+CD8+T cells-C1 cell clusters may be a risk factor for HCC patients. We also evaluated the prognosis of novel glycogen metabolizing cell clusters in different datasets in the immunotherapy TIDE database (Fig. 6F). In the LIRI and LICH population cohorts, different immunotherapeutic responses were seen for multiple glycogen metabolizing subtype cell clusters (*p* < 0.05) (Figs. 6G and 6H). IMvigor210 cohort included patients with metastatic uroepithelial carcinoma treated with anti-PD-L1 agents. In the IMvigor210 cohort, we found differences in PHLDA2+CAF-C1, IGF2+CAF-C2, INSR+CAF-C3, PPP1CB+CAF-C4, CALM3+ CD8+T_cells-C1, PPP1R2+CD8+T_cells-C4, PPP1CA+CD8+T cells-C5, and GF1+Mac-C1 cell clusters in patients with immunotherapeutic response (*p* < 0.05) (Fig. 6I). Finally, we performed KM prognostic analysis in the IMvigor210, LIRI, and LICH population cohorts and found that many novel glycogen metabolizing cell clusters are important for prognostic Overall Survival (OS) in the HCC population (Figs. 7–9). There is a close interrelationship between glucose metabolism and cells in the tumor microenvironment. In the tumor microenvironment, tumor cells exhibit higher sugar uptake and utilization compared to normal cells, producing energy and synthesizing substances through glycolytic pathways. In addition, the glycolytic metabolism of immune cells can affect their ability to kill and infiltrate tumor cells as well as the effectiveness of anti-tumor immune response.

## Discussion

To date, several studies have shown a link between alterations in glucose metabolism and pathogenesis of HCC [34–38]. However, most people have generally only explored the mechanism of action of glycometabolism-related genes in individual tumor microenvironment cells of HCC. In the present study, we have explored for the first time a comprehensive TME mapping study of glycogen metabolism-related genes for HCC, while demonstrating the interaction pathways between multiple glycogen metabolism subtypes of cells. Multiple glycogen subtype cell clusters were found to be associated with the prognosis of HCC. This unique perspective provides a new approach to explore the mechanisms of glycogen metabolism in HCC.

Metabolic molecules have a profound impact on the immune environment, thereby influencing the advancement of the disease [6,7]. Tumor-associated fibroblasts (CAFs) are one of the most important components of the tumor microenvironment and play an essential role in the development of tumors. CAFs suppress immune cell function and promote tumor development by secreting various cytokines to exchange information with other stromal cells and tumor cells [39]. A study by Li et al. demonstrated a novel mechanism by which CAFs interact with tumor cells to inhibit T-cell infiltration and reduce the efficacy of PD-1 antibody drugs [40]. The expression of surface markers of different subtypes of CAFs varies. Currently, through the analysis of several tumors, the more widely used classical analyses are pan-myCAFs, pan-dCAFs, pan-iCAFs, pan-nCAFs, and pan-pCAFs [31]. In our study, PPP1CB+CAF-C4 cell clusters were found to exhibit more extensive cellular communication with endothelial cells. Enrichment analysis found that the PPP1CB+CAF-C4 cell clusters participated in multiple metabolic pathways such as glycerophospholipid metabolism, glycerolipid metabolism, inositol phosphate metabolism, nitrogen metabolism, steroid biosynthesis, and sulfur metabolism. In addition, multiple transcription factor activities (such as JUNB, SOX17, and BCL5B,) were highly expressed in this cell cluster. Highly expressed PPP1CB+CAF-C4 cell clusters in HCC patients have a poor prognosis. Following tissue injury, mesenchymal cells undergo significant metabolic changes to promote energy-consuming cellular functions including proliferation and protein synthesis. Activation of fibroblasts increases glycolytic enzymes, such as hexokinase 2 and lactate dehydrogenase, which in turn promote cell proliferation and collagen synthesis [41]. Therefore, we speculate that glycogen-metabolized CAFs may have immunosuppressive interactions with tumor cells to promote tumor progression.

The microenvironment of liver tumors typically contains various cellular subsets of the innate immune system, such as macrophages, neutrophils, and natural killer (NK) cells, as well as T and B lymphocytes, which are central components of adaptive immunity [42]. In HCC patients, immune cells are not only responsible for anti-tumor response, but also contribute to cancer transformation, especially CD8 + T cells [43]. During infection with HBV or HCV, virus-specific CD8 + T cells are responsible for clearing the virus; however, these cells are somehow depleted and immunosuppressed by the liver environment and thus unable to eliminate the pathogen, leading to liver cancer [44]. Our study found that CALM3+CD8+ T cells-C1 cell clusters accounted for most of the CD8+ T cell population. CALM3+CD8+ T cells-C1 was found to be highly expressed in CD8+ T cell exhaustion. Therefore, it may be inferred that CALM3 inhibits tumor immunity by inhibiting CD8+T cells through glucose metabolism, thus leading to poor prognosis of HCC with high expression of CALM3+CD8+ T cells-C1 cell clusters. CALM3+CD8+ T cells-C1 cells are highly expressed in most metabolic signaling pathways, so glycogen metabolism may be one of the prognostic factors affecting patients with liver cancer. CALM3 gene encodes a member of a family of proteins that bind calcium and function as enzymatic cofactors. The activity of this protein is important in the regulation of the cell cycle and cytoplasmic division. Multiple selectively spliced transcript variants have been observed on this gene [45]. Meanwhile, CALM3 gene was involved in the progression of several diseases, such as cardiomyopathy, ventricular tachycardia, and long QT syndrome [45–48]. Although all these diseases were non-neoplastic, it can be speculated that CALM3 gene may be involved in other important metabolic processes.

To visualize the atlas in detail, we then investigated the glycogen metabolism atlas of B cell and macrophage cell clusters in the TME of HCC. Ding et al. and Yeung et al. reported that accumulation of tumor-associated macrophages was common in the liver of HCC patients and that macrophage numbers were associated with HCC progression and poor prognosis [49,50]. Phagocytes can differentiate into classical (M1) and other (M2) activated polar cells depending on different environmental conditions. Typically, M1 macrophages play a pro-inflammatory role by expressing nitric oxide synthase (iNOS), while M2 macrophages express anti-inflammatory cytokines, such as IL-10, to promote tumor progression and metastasis. Our study found that the majority of glycogen-metabolizing macrophage clusters in HCC are highly expressed in the M1 type. In contrast to the way monocytes and macrophages are recruited to tissue damage areas, fibrogenic macrophages usually coordinate angiogenesis through a series of interactions with fibroblasts, which are the main cellular source of pathological ECM deposition in the process of fibrosis [51–54]. For example, recent studies have shown that amphoteric modulins derived from macrophages induce mesenchymal stromal cells to differentiate into myofibroblasts through integrin-AV-mediated transforming growth factor activation [55]. Previous work has shown that proximity is crucial to crosstalk between macrophages and contractile fibroblasts [51,52,56]. Prognostic analysis revealed that IGF1+Mac-C1 cell clusters were highly expressed in HCC patients with good prognosis. This suggests that glycogen-metabolizing subtype cells may contribute to HCC to inhibit tumor metabolism, progression, and metastasis.

To analyze the complex heterogeneity of glycolytic genes in TME of HCC, we comprehensively summarized the relationship between the scores of these subclusters with prognosis and immune response from a large publicly available RNA-seq cohort. It was evident that the prognosis of HCC patients with TME cell clusters of glycolytic subtypes varies dramatically and particularly for CAFs and macrophages, revealing a critical role of TME glycolysis in HCC patients for future research.

Here, we performed a preliminary glycolytic mapping study of TME cells in HCC. However, a main limitation is the relatively small sample size; more samples are needed for the validation of our analysis. Compared to bulk RNA-seq, scRNA-seq of some glycolytic genes in HCC was usually smaller and had more zero observations, which may have led to bias in the clustering approach in our study. Nevertheless, the scRNA-seq analysis provided us with a new perspective by elucidating the characteristics of glycolytic genes in various TME monocytes to reduce HCC tumor heterogeneity, which is a critical step forward clinically.

## Conclusion

We identified for the first time the molecular profiles of TME cell subtypes modified by glycogen metabolism in HCC by single-cell sequencing analysis, revealing the role of glycogen metabolism-mediated intercellular communication in the tumor microenvironment on tumor growth regulation and anti-tumor immune modulation.

## Supplementary Materials

**Supplement Figure 1:**
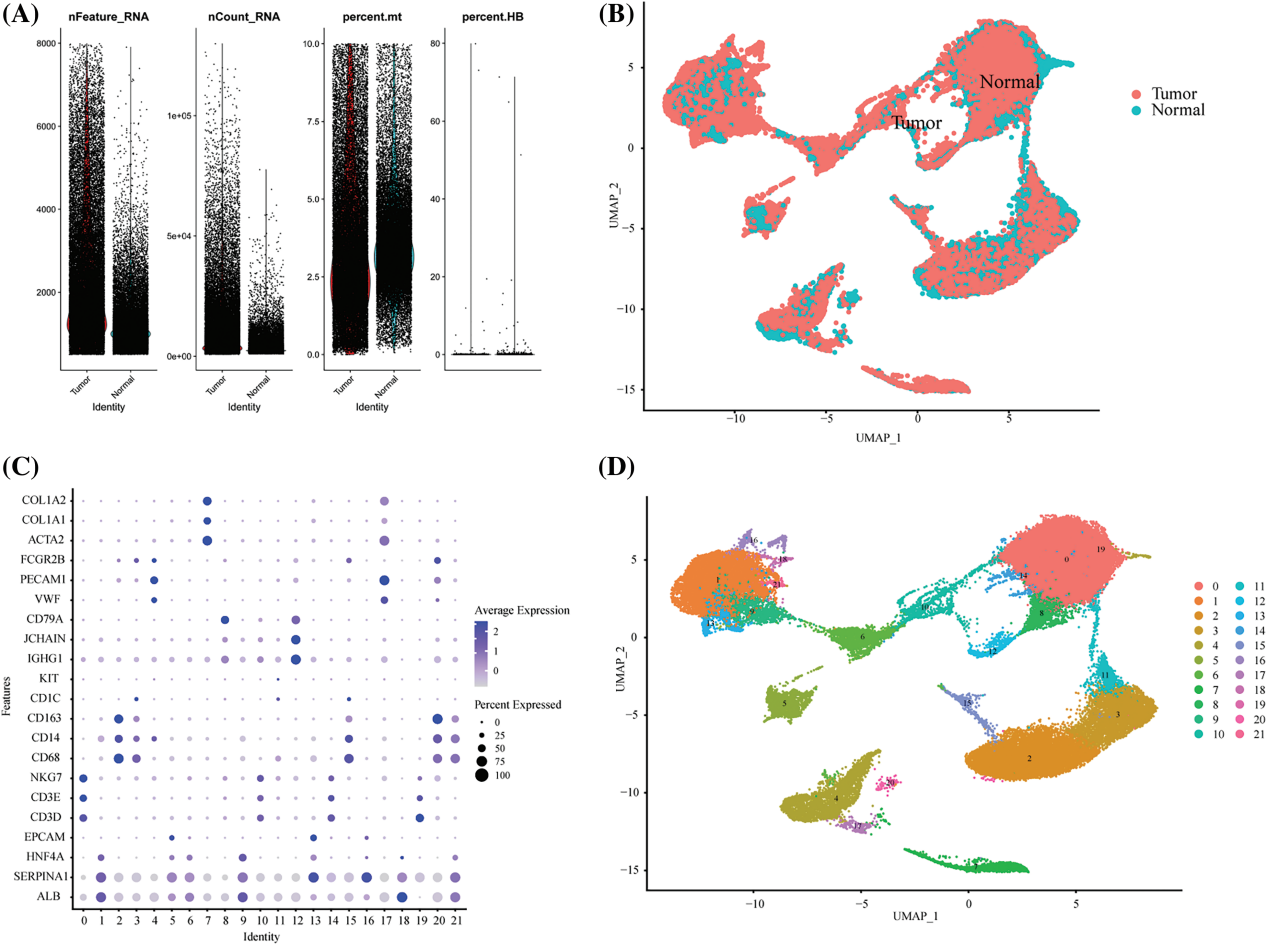
Single-cell quality control process. (A) The violin picture showed the number of filtered cells. (B) UMAP showed the distribution of tumors and normal tissues after harmony. (C) The expression of cell marker genes in different cell clusters. (D) The UMAP diagram showed the distribution of cell clusters in different groups before labeling.

## Data Availability

The datasets in all articles were downloaded from the Gene Expression Omnibus (GEO) database (https://www.ncbi.nlm.nih.gov/geo/info/overview.html).

## Abbreviations

NMF: Nonnegative matrix factorization
HCC: Hepatocellular carcinoma
ICGC: International cancer genome consortium
TCGA: The cancer genome atlas
